# Progress and strength of response against noncommunicable diseases in the US-affiliated Pacific Island jurisdictions, 2010–2021

**DOI:** 10.5365/wpsar.2022.13.1.843

**Published:** 2022-02-18

**Authors:** A Mark Durand, Haley L Cash, Zoe Durand

**Affiliations:** a Pacific Islands Health Officers Association, Honolulu, HI, United States of America.; b Tennessee Department of Health, Nashville, TN, United States of America.

## Abstract

**Objective:**

To determine the effectiveness of the response to the 2010 declared regional noncommunicable diseases (NCDs) emergency in nine US-affiliated Pacific Island jurisdictions.

**Methods:**

Vital statistics and risk prevalence surveys were retrospectively reviewed using 14 standardized NCD risk, prevalence and death rate indicators to measure changes in health status over time. NCD risk and prevalence change scores were derived from subsets of these indicators, and NCD composite death rates were examined. An NCD strength-of-intervention score derived from a standardized regional monitoring tool provided measures for assessing responses aimed at curbing risk factors, prevalence and death rates. Associations between the strength-of-intervention score and changes in health status were examined.

**Results:**

Pairs of values were available for 97 of 126 individual comparisons for 14 core indicators in nine jurisdictions. The composite mean prevalence of all risk factors across the jurisdictions between baseline and follow-up (26.7% versus 24.3%, *P* = 0.34) and the composite mean diabetes and hypertension prevalence (28.3% versus 28.2%, *P* = 0.98) were unchanged, while NCD death rates increased (483.0 versus 521.9 per 100 000 per year, *P* < 0.01). The composite strength-of-intervention score for the region was 37.2%. Higher strength-of-intervention scores were associated with improvements in health indicators.

**Discussion:**

Despite some improvements in selected NCD indicators at the jurisdiction level, there was no significant overall change in the prevalence of risk factors, diabetes and hypertension, and death rates have continued to increase since the NCD emergency declaration. However, the adoption of public sector NCD interventions was associated with improvements in health indicators.

Globally, the Pacific Islands are largely considered to be among the regions most severely affected by noncommunicable diseases (NCDs). (*1*-*5*) In the Pacific, NCDs are fuelled by several behavioural risk factors, including substantial rates of tobacco use and problem alcohol drinking, and (especially) patterns of diet and physical activity that result in a high prevalence of obesity. (*6*-*9*) In 2010, the Pacific Islands Health Officers Association (PIHOA), comprising the heads of health in the US-affiliated Pacific Islands (USAPI) – American Samoa, Commonwealth of the Northern Mariana Islands, Guam, Republic of Palau, Republic of the Marshall Islands, and four states of the Federated States of Micronesia (Chuuk, Kosrae, Pohnpei and Yap) – issued a regional declaration of health emergency for NCDs. The declaration called for an intensified response, guided by data. (*10*) Shortly after the declaration, PIHOA convened technical working groups to develop a framework for tracking the progress of the NCD emergency, and for monitoring the response to NCDs and the impact of the declaration. With this effort, the USAPI became the first international group to recognize and organize a systematic response to NCDs. The surveillance framework includes the following indicators with standardized data definitions: youth and adult tobacco smoking and tobacco chewing; youth alcohol use and adult binge drinking; youth and adult overweight and obesity; adult diabetes and hypertension prevalence; and cause-specific death rates for cancer, cardiovascular disease, chronic lung disease and diabetes (Unpublished document: USAPI NCD Core Monitoring & Surveillance Framework. Honolulu, Hawaii: Pacific Islands Health Officers Association; 2012. Available on request). In contrast with infectious disease surveillance, which largely depends on tracking incident cases, NCD surveillance depends on conducting periodic, population-based surveys. These must have consistent survey questions, physical measurement methods, age groups and sampling across jurisdictions and over time. Although difficult to deploy repeatedly and consistently, population-based surveys give a much clearer picture than institution-based incidence data of the burden of NCDs in populations. The use of a predetermined, balanced set of risk, disease prevalence and death indicators across multiple jurisdictions for the past decade is a major strength of this surveillance system. The framework aligns closely with the subsequently released World Health Organization (WHO) monitoring framework, although the USAPI framework measures not just the core indicators recommended by WHO but also youth risk factors. (*11*)

The USAPI framework called for monitoring the uptake of a specific suite of NCD policy measures developed by a Pacific-wide technical working group, the Pacific Monitoring Alliance for NCD Action (MANA). The MANA coordination team includes NCD policy experts from the Pacific Community (SPC), WHO, PIHOA and the Pacific Centre for Prevention of Obesity at Fiji National University. Indicator definitions and assessment criteria were developed, refined and piloted by the coordination team, and endorsed by the Pacific Heads of Health and Pacific Health Ministers groups in 2017, with the inaugural assessment report released in 2018. Progress is tracked via annual country-based assessments and reported on a MANA dashboard, which is updated every 1–3 years by each member jurisdiction, with assistance from MANA technical partners.

The MANA dashboard comprises 31 NCD interventions covering six categories including preventive policies for tobacco, policies for alcohol, policies for food environments and physical activity, health services system changes, leadership and governance structures, and surveillance and monitoring systems. (*12*, *13*) The use of predefined measures for both health status and response across multiple jurisdictions presents an opportunity to systematically examine progress in the fight against NCDs.

In this report, we examine progress in the USAPI jurisdictions by examining the change in health status indicators in the USAPI framework in the 10 years since the emergency declaration. We also look at the strength of the response against NCDs in the USAPI (reflected as intervention scores derived from the MANA dashboards from USAPI jurisdictions) and examine the relationship between the strength of intervention and changes in population health status.

## Methods

In this study, risk, disease prevalence and death rates were collected for each USAPI jurisdiction using historical sources dating back to 2000. Sources included surveys from the WHO STEPwise Approach to NCD Risk Factor Surveillance (STEPS); customized, PIHOA-facilitated, community-based hybrid NCD adult surveys; US Centers for Disease Control and Prevention (CDC) Behavioural Risk Factor Surveillance System surveys; CDC Youth Risk Behaviour Surveillance surveys; PIHOA-facilitated, customized Rapid High School Youth Surveys; the US National Center for Health Statistics mortality databases in the three US territories (Guam, the Commonwealth of the Northern Mariana Islands and American Samoa); and jurisdiction vital statistics office databases for non-territory USAPI (the Freely Associated States of Palau, Marshall Islands and the four states of the Federated States of Micronesia). Convenience surveys were excluded. Prevalence estimates were compared from surveys that used consistent sampling, collection methods and survey questions as set forth in the USAPI NCD Core Surveillance Framework and Data Dictionary.

NCD premature mortality rates were 5-year running averages, for those aged 30–69 years, age-adjusted to the WHO 2000–2025 standard population. (*14*) Prevalence of overweight or obesity, diabetes and hypertension were reported only from studies that included physical measurements of height, weight, blood pressure and fasting blood sugars, omitting those that relied solely on self-reported disease status.

The date of the PIHOA emergency declaration, May 2010, was considered the reference date for baseline measures. For each jurisdiction, the earliest available data point between 2010 and 2013 was used as the baseline value for each indicator, whereas the most recent available data point from 2015 to the present was considered to represent “recent status.” If no baseline data point was available between 2010 and 2013, we used data from surveys conducted before 2010.

A composite indicator (the NCD risk and disease prevalence change score) was calculated as the average change from baseline in the prevalence of all risk factors, diabetes and hypertension. In addition, category-specific change scores were produced by averaging the change in prevalence for all indicators within each of the following categories: tobacco, alcohol, nutrition and physical activity, and diabetes and hypertension. A composite NCD death rate indicator was calculated as the sum of death rates for cardiovascular disease, cancer, diabetes and chronic lung disease. These composite indicators were used to assess overall changes from baseline for each category, by jurisdiction and for the region as a whole (e.g. the average of all baseline tobacco use prevalence values for youth and adults across the region was compared with the average of values at follow-up, to assess overall tobacco trends). The scores were not adjusted for the differing population sizes of the jurisdictions; they represent the average of changes that each individual jurisdiction has managed to achieve, and do not measure the true changes in prevalence of the USAPI population as a whole. Only data points having both baseline and follow-up values were included in composite indicator calculations. Confidence intervals (CI) for NCD risk and disease prevalence change score results were calculated using *t*-tests at a 95% confidence level. Changes in death rates were assessed using Z-scores.

The strength of the NCD response was gauged using strength-of-intervention scores derived from the MANA dashboard. Each intervention item in the dashboard was awarded between 0 and 5 points, based on the strength of the intervention. Intervention scores were calculated as the current percentage of maximum possible points awarded for a group of response items, and were stratified by intervention category and by jurisdiction.

Intervention scores range from 0% (no actions taken) to 100% (all recommended interventions are implemented). For example, the regional tobacco intervention score is the sum of the points for all tobacco items across all nine jurisdictions, divided by the number of points possible × 100%, whereas the overall intervention score is the sum of the points for all intervention items across all nine jurisdictions divided by the number of points possible × 100%.

The relation between strength-of-intervention and change in health status indicators was explored using linear regression, with the intervention score for each category of intervention (tobacco, alcohol, nutrition or physical activity, and health services) as the independent variable. The log of the relative change from baseline of the corresponding health status indicators (i.e. for tobacco, alcohol, overweight or obesity, and NCD death rates) was used as the dependent variable (with tobacco intervention items linked to tobacco indicators, alcohol items to alcohol indicators, nutrition or physical activity items linked to overweight and obesity indicators, and clinical health services linked to NCD death rates). For example, if baseline versus recent cigarette use prevalence is 50% versus 30%, the relative change is (0.30 – 0.50)/0.50 = –0.40.

The relationship between average intervention scores across all intervention categories (as the independent variable) and the log of the relative change of all health status indicators (as the dependent variable) was used to provide an overall picture of how well the nine jurisdictions were doing relative to one another. Log transformation of the relative change in health status indicators was employed to address skewness of the outcome data (skewness value = 3.69 for relative change in health status indicators versus –0.41 for log transformed data).

## Results

Risk factors and disease prevalence, and their changes from baseline varied considerably across jurisdictions (**Tables 1, 2**). The NCD risk and disease prevalence change scores for each indicator category (95% CI) were as follows: alcohol –4.2% (–7.7, –0.7), tobacco –2.4% (–5.3, 0.0), overweight and obesity +1.5% (–4.5, +7.4), and diabetes and hypertension –0.4% (–5.1, +4.3). Negative scores indicate improvement and positive scores worsening of health status over time.

Death rates also varied substantially across jurisdictions (**Table 3**). Composite premature NCD death rates (including deaths from cardiovascular disease, cancer, diabetes and chronic lung disease) for the region as a whole increased from a baseline of 483.0 to 521.9 per 100 000 residents aged 30–69 years (*P* < 0.01).

### Baseline versus recent death rates

**Table 4** shows MANA intervention scores for tobacco, alcohol, overweight or obesity, and health services intervention categories by jurisdiction. The average overall NCD strength-of-intervention score for the region was 37.2%. There was considerable variation in the strength of intervention by category, from 24.9% for nutrition or physical activity to 48.1% for tobacco. The average composite strength-of-intervention score across intervention categories by jurisdiction also varied considerably, from 25.0% for the Republic of the Marshall Islands to 54.8% in Guam.

**Fig. 1:**
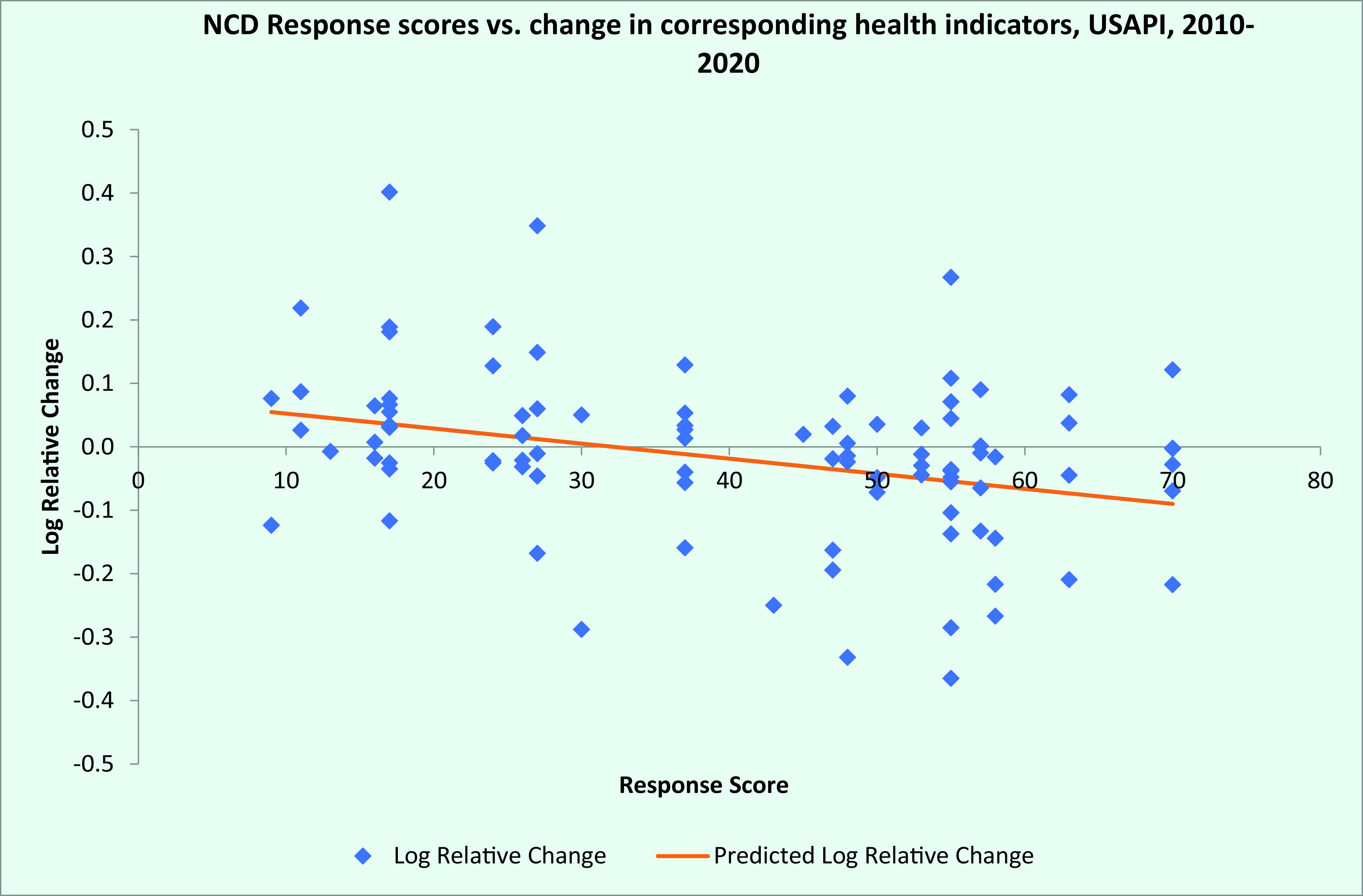
Noncommunicable disease intervention scores versus change in corresponding health indicators, US-affiliated Pacific Islands, 2010–2020

**Fig. 2:**
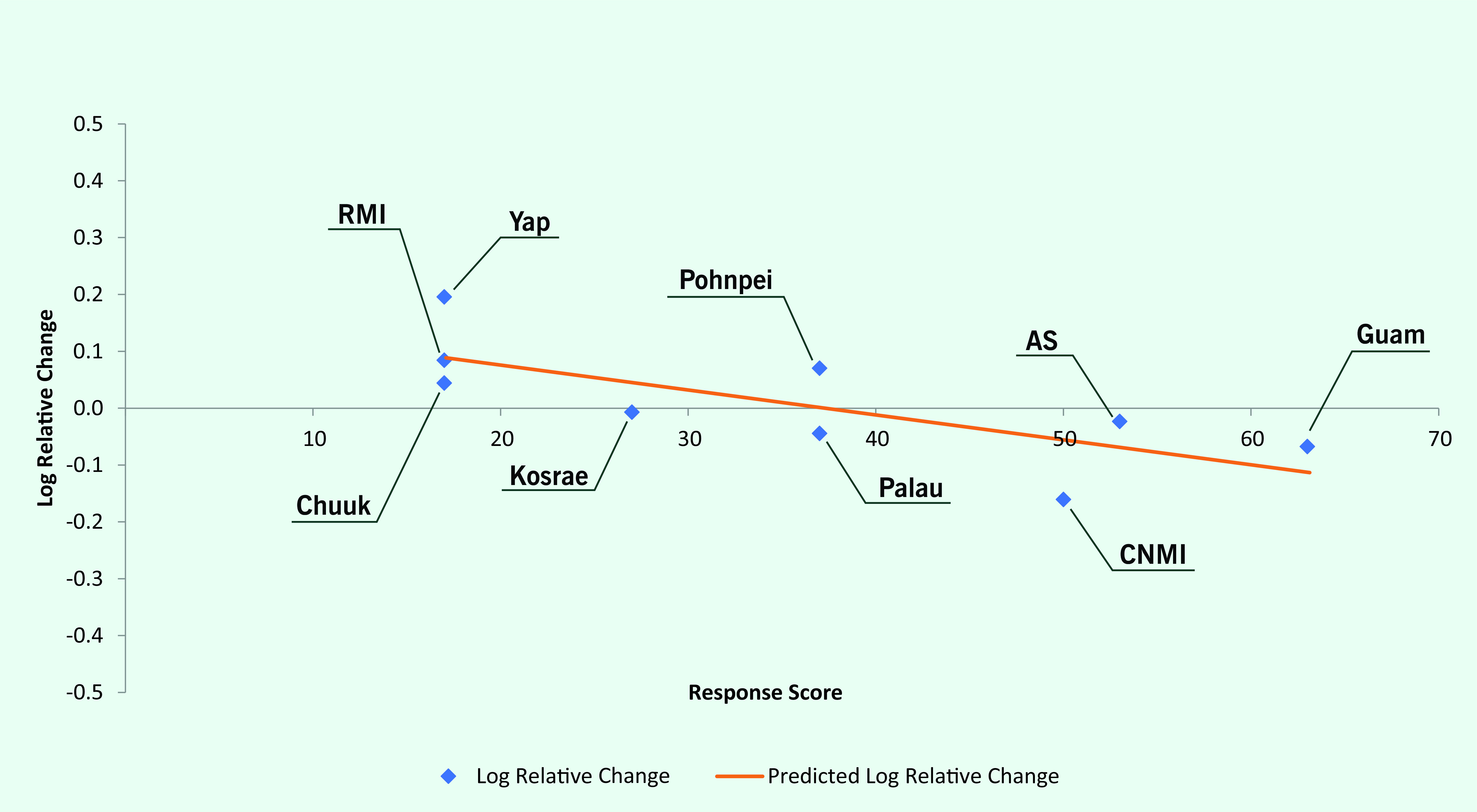
Composite jurisdiction noncommunicable disease intervention scores versus relative change in health indicators, US-affiliated Pacific Islands, 2010–2020

Log linear regression showed an overall negative relationship between response scores and the log of relative changes in health status indicators, with an R^2^ of 0.063 and regression line slope of –0.0024 (*P* = 0.01) (**Fig. 1**). This suggests an average improvement in related health status indicators of 2.7% for every 10% increase in the corresponding response index. Log linear regression also showed a negative relationship between average response scores by jurisdiction and the log of relative changes in health status indicators by jurisdiction (regression line slope = –0.0044; *P* = 0.02) (**Fig. 2**).

[**insert** Figure 1]

[**insert** Figure 2]

## Discussion

Through collective action, USAPI countries and territories have defined a consensus set of core NCD response measures and health status indicators, permitting a concerted approach to addressing the NCD crisis and to monitoring progress in the region.

This study shows that, since the NCD emergency declaration in 2010, there was no change in the composite mean prevalence of all risk factors across the USAPI jurisdictions between baseline and follow-up and the composite mean diabetes and hypertension prevalence, whereas NCD death rates significantly increased. There were some improvements in the prevalence of alcohol and tobacco use, and increases in obesity prevalence and NCD death rates. Given these results, it will be difficult to meet the United Nations Sustainable Development Goal 3.4: “By 2030, reduce by one third premature mortality from non-communicable diseases.” (*15*) NCD prevalence and death rates are largely the result of long-standing behaviours and they change relatively slowly in response to policy measures (although improvements in health services can improve death rates more rapidly). In contrast, risk factor prevalence changes more rapidly in response to effective policy measures; thus, the decline in some risk factors could presage future improvements in disease prevalence and mortality as their benefits accrue over time. The only jurisdiction to show a decrease in NCD death rates, the Northern Mariana Islands, has one of the highest intervention scores.

Across all jurisdictions, the strength of response score was 43%, indicating that many evidence-based interventions have not yet been implemented; most of these interventions are the province of policy-makers outside the health sector. As noted by others, strengthened multisectoral commitment is therefore a key to success. (*16*)

Our conclusions are subject to several limitations. First, various data points for either baseline or recent core health status indicators were not available for some jurisdictions. Timely, routine surveillance activities (youth school-based surveys, adult community-based surveys and analysis of vital statistics data) based on jurisdiction-level NCD monitoring and surveillance plans are needed to fill these gaps and provide a more complete picture of the ongoing NCD emergency. Second, although we would have liked to use 2010 baseline values and recent data points for each indicator, the collection years and time span between the baseline and recent data points vary among indicators and jurisdictions, introducing some uncertainty in assessing progress.

Deficiencies in the completeness and accuracy of mortality reporting that have been observed in the region may also have affected our findings, while out-migration from several of the jurisdictions since censuses were last conducted (between 2010 and 2015) may also have affected mortality rate estimates. (*17*) In addition, it would be useful to track the exact dates of initiation of interventions. However, some interventions (e.g. tobacco and alcohol tax increases) are introduced in phases and implementation times for others are unclear. Finally, the numerical scoring of ordinal values used in the NCD intervention scores may compromise precision in these measures (since the expected impact from each additional point within an intervention and the expected impact of each point from one intervention to the next may not be constant).

In summary, declaring a regional emergency for NCDs in USAPI has stimulated the development of standardized frameworks for NCD surveillance and response. Although surveillance for NCDs is challenging and additional investments are needed to address gaps and assure rigorous conduct of surveys, existing data do yield a detailed picture of progress over the past 10 years. Some progress has been made towards better control of alcohol and tobacco, but there is little change in other measures of health. The evidence supports the effectiveness of policy and health system interventions in the context of the Pacific Islands; however, many of the recommended NCD interventions have not been adopted, especially in the most affected areas (geographical and risk factors). A renewed commitment to adopt these measures is needed to decisively turn the tide of NCDs in the region.

### Public health implications

Agreement across countries and territories on a core set of predefined NCD-related response measures and health status indicators enables a systematic approach to monitoring the response to the NCD crisis and resulting changes in population health status. The provision of such high-quality feedback is useful for strategic planning and evaluation for public health practitioners, technical assistance agencies and policy-makers. The discrete groupings and modest population sizes within multiple jurisdictions and the ability to track the impact of interventions make the USAPI an attractive setting for testing innovative approaches to the NCD crisis.
